# Preparation and Properties of Glycerohydrogels Based on Silicon Tetraglycerolate, Chitosan Hydrochloride and Glucomannan

**DOI:** 10.3390/gels11020103

**Published:** 2025-02-02

**Authors:** Sergei L. Shmakov, Olga S. Ushakova, Marina A. Kalinicheva, Anna B. Shipovskaya

**Affiliations:** Chair of Polymers, Institute of Chemistry, Saratov State University, 83 Astrakhanskaya St., 410012 Saratov, Russia; olgakol4ina777@yandex.ru (O.S.U.); marinka2501@gmail.com (M.A.K.); shipovskayaab@yandex.ru (A.B.S.)

**Keywords:** glycerohydrogel, gelation, chitosan, glucomannan, silicon glycerolate, sodium tetraborate, aminocaproic acid, bioadhesive properties

## Abstract

Glycerohydrogels based on silicon glycerolate, chitosan (CS) and polyvinyl alcohol (PVA) are widely studied for use in biomedical applications. In line with the general trend of replacing synthetic polymers with natural ones in such compositions, it would be of interest to replace PVA with the polysaccharide glucomannan (GM), as well as to introduce functional additives to impart the desired properties, including gelation time, to the final hydrogel. In this work, a comprehensive study of the preparation conditions and properties of glycerohydrogels based on silicon tetraglycerolate, chitosan hydrochloride (CS·HCl) and GM was carried out. Viscometry was used to assess the conformational state of CS·HCl and GM macromolecules, and their associates in solution before gelation. Gelation was studied using the vessel inversion method. The mucoadhesive and the dermoadhesive properties of the glycerohydrogels obtained were assessed using the tearing off method from the model substrates simulating mucous and dermal tissues. The conformational state of the individual polymers and their mixed associates in solution before gelation was estimated; the intrinsic viscosity and the hydrodynamic radius of the macromolecular coils were calculated. The influence of various factors (addition of ε-aminocaproic and hydrochloric acids, sodium chloride, hydroxide and tetraborate to vary the acidity and ionic strength of the medium, as well as temperature) and the molecular weight of chitosan on the gelation time was studied. The gelation time achieved was less than 2 min, which is promising in practical terms, i.e., for creating liquid plasters. Our best samples are not inferior to the commercial preparation “Metrogyl Denta”^®^ in terms of tearing force during mucoadhesion and dermoadhesion at short gelation times. Thus, the glycerohydrogels synthesized by us and based on silicon tetraglycerolate, CS·HCl and GM could find usage in new biopharmaceutical and biomedical applications.

## 1. Introduction

The design of biocompatible, biodegradable and environmentally friendly hydrogel systems is one of the urgent tasks in the field of polymer biomaterials science [1,2,3,4]. The base polymers for such systems are usually various natural polysaccharides, the aminopolysaccharide CS (Figure S1a) being especially promising [5,6,7]. The variety of biologically valuable properties of this polymer determines the possibility of obtaining various hydrogel materials on its basis with both improved and new properties. In slightly acidic and close to neutral aqueous media, CS forms a gel due to ionic and hydrogen bonding between its macromolecules. Such hydrogels, however, have low strength and are kinetically unstable. To enhance gelation, bifunctional cross-linking agents are added to CS, e.g., glutaraldehyde and other dialdehydes, whose side condensation products, unfortunately, are toxic. In addition, the pH of the chitosan-containing hydrogels is usually 3.5 or less, which limits their use in medical and biological applications. Therefore, it is important to search for new approaches and methods for carrying out the gelation process in CS solutions which would ensure the production of a kinetically stable gel form of the chitosan-containing system with a shift in pH towards neutrality and maintaining its biological activity.

For biomedical gels, which are required to biodegrade within a specified period (after performing their function, say, as a scaffold or liquid patch), physical rather than chemical cross-linking of macromolecules, weaker cross-links between them, are desirable. Biodegradation is achieved primarily by enzymatic hydrolysis of glycosidic bonds, while covalent cross-linking bonds preserve all monomer CS units within a common 3D (albeit weakened) network for a long time. Therefore, physical gelation of CS receives much attention in the literature (e.g., review [8]).

Physical gelation can be achieved, for example, by adding propanediol to a CS solution in acetic acid with evaporation of the solvent and neutralization with alkali [9]. The hydrogels obtained by the authors contained only CS and water after washing. A substance with a weakly alkaline reaction (to avoid precipitation of CS) can be added, such as sodium bicarbonate [10].

Ammonia is one of the weak bases used for physical CS gelation. Various methods have been developed to address its slow introduction into the system, e.g., Wlodarczyk et al. [11] used ammonia released during the enzymatic hydrolysis of urea. When tracing pH changes over time, two gelation points were established. Gelation time was determined rheologically (equality of the elastic and viscosity moduli, pH 6.4), whilst advanced gelation time (longer) was determined by inverting the vessel (pH 7.4). Montembault et al. [12] placed the vessel with a CS solution in acetic acid in an ammonia atmosphere, and the gelation point was determined rheometrically (equality of the storage and loss moduli). The effect of the CS acetylation degree on the gelation time was studied. The authors concluded that the acetamide groups of CS were involved in the gelation process via hydrophobic interactions. Ammonia can be introduced from either the gas or the liquid phase [13] when it penetrates from top to bottom.

β-glycerophosphate (sodium salt, weak base—p*K*_a_ 6.65 at 25 °C) is popular for physical cross-linking of chitosan macromolecules. Chenite et al. [14] brought a CS solution to physiological pH with β-glycerophosphate without gelation within 5–15 °C, then heated it to form a gel. A similar method was used by Cho et al. [15]. The authors found that by adding β-glycerophosphate, the pH of a CS solution can be increased to almost neutral without gelation. Ahmadi and de Bruijn [16] observed gelation in the same way at human body temperature (2–10 min), i.e., the gel was heat-sensitive. The thermal sensitivity of such gels was confirmed by Ganji et al. [17]. The gelation time at 37 °C decreased with increasing component concentrations, temperature, and CS deacetylation degree. The sol–gel transition time was estimated by inverting the vials horizontally every minute.

The gelation time by this method can be regulated by adding excipients—hydroxypropyl methyl cellulose, polyethylene glycol with two molecular weights, and poloxamer [18]. It should be noted that in this case, not only hydrogen bonds involving deprotonated amino groups play a role but also screening of the electrostatic repulsion of protonated amino groups, ionotropic cross-linking, and the hydrophobic effect.

Sodium tripolyphosphate exhibits ionotropic cross-linking of chitosan macromolecules at low pH (at high pH, it occurs via the deprotonation mechanism) [19]. Hamdi et al. [20] added poloxamer to the system and found that the gelation temperature is symbatic to the CS acetylation degree.

ε-Aminocaproic acid (AmA) is used as an additive to CS solutions, which are then gelled. Du et al. [21] added AmA to a liquid plaster based on poloxamers with chitosan to stop bleeding, since it effectively inhibits fibrin dissolution (proteinase inhibitor). Park et al. [22] introduced AmA into dental chitosan particles to stop bleeding as well. It is even grafted onto CS macromolecules to increase the solubility of the polymer in water [23].

Combinations of physical cross-linking agents such as NaOH and phosphate buffers are also used [24]. Physical cross-linking means that NaOH or a phosphate buffer deprotonates the protonated amino groups of chitosan, as a result of which their ability to form hydrogen bonds returns. Such bonds, formed between different macromolecules, provide ionotropic gelation with no formation of covalent bonds. The use of polyelectrolyte complexes with anionic polysaccharides is also promising [25].

Despite the high biopotential of chitosan salts with organic biologically active acids for obtaining hydrogels [26,27], in our opinion, such a simple inorganic acid as a hydrochloric one and its salt with CS (chitosan hydrochloride) should not be neglected. This acid is still being studied as a solvent for this polymer [28], e.g., Qiao et al. [29] compared several CS solvent acids and concluded that it is CS·HCl that exhibits the highest degree of crystallinity in the solid state. Minh et al. [30] proposed a new method for obtaining CS·HCl by treating chitosan in the solid phase with gaseous hydrogen chloride and noted a number of advantages of the resulting product compared to that obtained in a liquid medium. Hydrochloric acid was used to hydrolyze chitosan [31] to obtain chitosan oligomers with more pronounced biological properties than high-molecular CS. Wu and Zhang [32] used concentrated hydrochloric acid, which was compared with diluted one. CS·HCl exhibits mucoadhesive properties, increased permeability through epithelial tissues and effective antimicrobial activity [33,34,35]. It was also established that the biological activity of CS ascorbate hydrochloride (a complex salt of CS·HCl with ascorbic acid) is significantly higher than that of CS ascorbate [26,36]. All this suggests that hydrochloric acid has not yet exhausted its potential as a solvent for CS.

In addition to purely chitosan hydrogels, hybrid ones are also obtained using biomimetic sol–gel technology, one of the relatively new and promising areas for obtaining biocompatible materials [37]. A number of publications [38,39,40] are devoted to the sol–gel synthesis of organo-inorganic polyfunctional hybrid structures, including those based on chitosan and silicon. To improve the stability of the material in the chemically active environment of the human body, and to increase bioactivity and maintain biocompatibility, pharmacologically active silicon-containing precursors, in particular, silicon tetraglycerolate, are used [41]. In a number of works [26,41,42,43,44], we tested the biomimetic sol–gel synthesis of silicon–chitosan-containing glycerohydrogels using glycerol solutions of silicon tetraglycerolate (Si(OGly)_4_) and aqueous solutions of chitosan salts with biologically active organic acids (glycolic, ascorbic, and aspartic). It was found that chitosan accelerates the gelation of silicon tetraglycerolate in slightly acidic media, while in more acidic media, the process rate curve passes through a maximum at a certain concentration of CS, which could be due to different mechanisms of the silanol condensation reaction before and after the isoelectric point.

It is known [45] that macromolecules with –OH groups (e.g., polysaccharides and PVA) significantly accelerate the kinetics of the sol–gel process of Si precursors and promote the polycondensation of silanol groups. In template sol–gel synthesis using PVA, CS ascorbate and Si(OGly)_4_, it is possible to obtain not only bulk Si–chitosan-containing gel monoliths but also thin-film glycerohydrogel plates which exhibit characteristic features of soft elastic materials, including congruence to a surface with a complex relief [26,43]. However, despite the bioinertness of PVA and its inclusion in the list of biodegradable plastics, the synthetic nature of this polymer predetermines the fundamental differences in structural and functional properties from biopolymers. In this connection, it would be of interest to introduce into the gelling system, along with CS, a neutral polysaccharide with hydroxyl groups, e.g., glucomannan (Figure S1b). It is a good gelling agent for aqueous solutions [46]. In addition, GM is compatible with other polysaccharides, non-toxic, biodegradable, and has a number of other valuable properties for biopharmaceutical and biomedical applications [47,48,49]. Like PVA, GM in combination with CS aspartate and Si(OGly)_4_ forms sol–gel plates, the supramolecular structure of which is represented by a system of interpenetrating spatial networks of organic and inorganic nature in a water–glycerol medium [44]. The organic network is formed by a physical gel of the polysaccharides (GM and CS), whose –OH groups serve as a template for the condensation of Si–OH groups into disiloxane groupings Si–O–Si and the subsequent synthesis of an inorganic chemical network of silicon polyolate. It seems that the observed synergistic effects could be significantly enhanced by using mixtures of GM with CS·HCl. This salt form of CS has increased solubility in water [28], which allows the use of aqueous solutions of GM + CS·HCl mixtures without the use of acidic CS solvents. This expands the areas of possible medical application of such hydrogels. It also seems that the introduction of various additives into the gelling composition, e.g., those regulating the pH of the medium and/or the protonation degree of CS amino groups, salts–electrolytes of the lyotropic Hofmeister series to accelerate the gelation of Si(OGly)_4_ or to cross-link the diol–diol functional groups of GM macrochains will promote both the structural compaction of the hydrogel network and the acceleration of gelation up to ultrafast (less than 2–3 min) sol–gel synthesis.

The aim of this work was to obtain glycerohydrogels based on silicon tetraglycerolate, chitosan hydrochloride, and glucomannan, and to study the effect of temperature, pH, and functional additives (ε-aminocaproic acid, sodium tetraborate (STB) and chloride) on the gelation time.

## 2. Results and Discussion

### 2.1. Viscometric Properties of Solutions of Glucomannan, Chitosan Hydrochloride, and Their Mixtures

**Figure 1:**
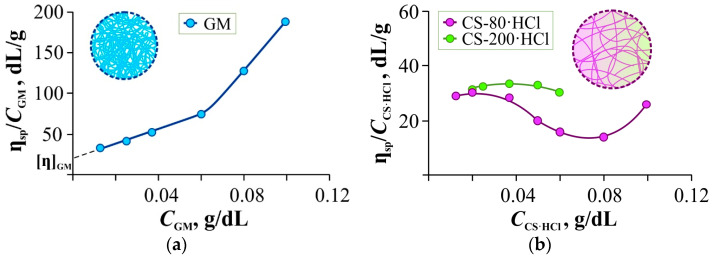

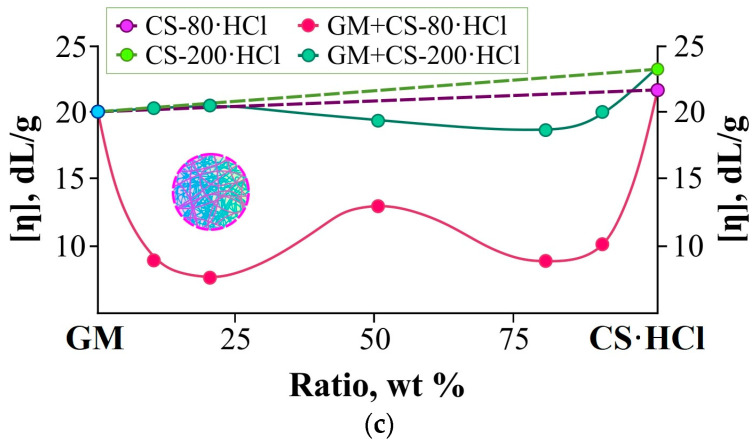
Viscometric properties of solutions of glucomannan, chitosan hydrochloride and their mixtures. (**a**,**b**) Concentration dependence of the reduced viscosity (η_sp_/*C*) of solutions of GM (**a**), CS-80·HCl and CS-200·HCl (**b**) (CS-200·HCl solutions of higher concentrations cannot be studied by capillary viscometry, since the solution flow time would be too long due to the high molecular weight of the polymer, which would lead to large errors in determining the reduced viscosity.) at 25 °C. (**c**) Diagram of the intrinsic viscosity of GM + CS-80(200)·HCl mixtures of different compositions, 25 °C; the dashed straight lines correspond to the additive values of intrinsic viscosity [η]. The insets show a schematic representation of the macromolecular coils of GM (**a**), CS-80(200)·HCl (**b**) and the associate GM + CS-80(200)·HCl (**c**).

In the first stage, the viscosity of the polymer solutions was measured as a function of concentration. Figure 1a,b shows the concentration dependences of the reduced viscosity of aqueous solutions of GM, CS-80·HCl, and CS-200·HCl (the numbers correspond to the viscosity-average molecular weight of the samples in kDa).

For the nonionic polysaccharide GM (Figure 1a), this dependence is linear (with a break point at ~0.06 g/dL) and allows estimating the intrinsic viscosity from the Huggins plot. For the ionogenic CS-80(200)·HCl (Figure 1b), due to dilution with water rather than with hydrochloric acid of the corresponding concentration, the curves show a clear polyelectrolyte effect. However, plotting the graphs in the Fuoss–Strauss coordinates *c*/η_sp_ vs. *c*^1/2^ [50] yielded no linear dependence. Therefore, the formula (∂ln η_rel_/∂*C*)*_C_*_→0_ [51] was used to estimate [η]. A graphical estimate of the intrinsic viscosity using this method is given in Figure S2. The intrinsic viscosities and effective hydrodynamic radii of the studied polymers are summarized in Table 1.

It is noteworthy that the differences in [η] are small between the chitosan samples with quite different molecular weights. This may be due to the higher content of bound hydrochloric acid in the commercial sample CS-80·HCl (9.7 wt.% Cl) compared to the laboratory sample CS-200·HCl (7.6 wt.% Cl). Because of this, the coils of longer macromolecules are compressed more strongly and thus differ little in hydrodynamic volume from the swollen coils of shorter macromolecules.

Figure 1c shows the effect of the different ratios of polymers on the intrinsic viscosity of the mixed polymer solution. The straight lines indicate additivity (the contribution of each polymer to the intrinsic viscosity, taking into account its share in the mixture, is summed up). As can be seen from this figure, negative deviations from additivity are observed in both cases, however, in the case of high-molecular-weight CS-200·HCl they are significantly smaller than in the case of low-molecular-weight CS-80·HCl. This can be explained by the fact that the GM and CS-80(200)·HCl macromolecules form mixed coil complexes with hydrogen bonds between themselves, which are denser and more compact than the macromolecular coils of the polymers individually. The shorter the chitosan macromolecule, the easier it is for it to form such a mixed coil and, consequently, the greater the negative deviation from additivity will be.

It should be noted that the compaction of polymer coils during gelation could facilitate the formation of strong intermolecular bonds of both localized and non-localized types since the interaction of not individual chains, but aggregates of molecules plays a significant role in the formation of the spatial network.

### 2.2. Effect of Functional Additives on the Gelation Time of the System Based on Silicon Tetraglycerolate, Chitosan Hydrochloride and Glucomannan

**Figure 2:**
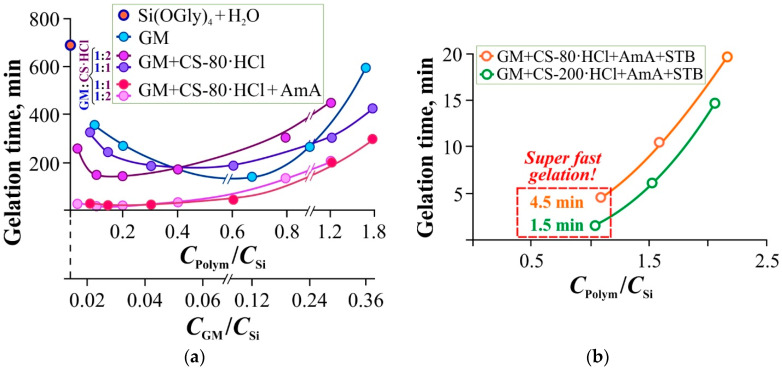
Dependence of the gelation time of the systems on the *C*_Polym_/*C*_Si_ ratio (*C*_GM_/*C*_Si_) for: (**a**) GM (the lower abscissa axis), GM + CS-80·HCl of 1:1 and 1:2 wt.% composition with and without the addition of AmA (the higher abscissa axis); (**b**) GM + CS-80(200)·HCl + AmA with the addition of STB (Systems with STB and without AmA were studied but are not included in our manuscript because without the addition of AmA the pH of the formed hydrogels is highly acidic, which is not suitable for biomedical applications), 25 °C. The systems with ultra-fast gelation (**b**) were then used to evaluate the mucoadhesive and dermoadhesive properties. The *C*_Polym_ values correspond to the total concentration of GM + CS-80(200)·HCl in the gelation system, *C*_Si_ is the concentration of Si(OGly)_4_. The absolute concentrations of the polymers in the mixture and their values recalculated to the silicon concentration are given in Tables S3-1 and S3-2. Here, in Figure 2a and further in Figure 4, the gelation time of Si(OGly)_4_ in an aqueous medium is indicated on the ordinate axis, *C*_Si_ = 47.0 wt.%.

Figure 2 shows the dependence of the gelation time of the systems based on glycerol solutions of Si(OGly)_4_ and aqueous solutions of GM, as well as mixed systems GM + CS-80(200)·HCl on the template/precursor weight ratio (*C*_GM_/*C*_Si_, *C*_Polym_/*C*_Si_, where Polym = GM + CS·HCl). When adding GM to the system, due to its high viscosity (a high molecular weight), the concentration of the latter was chosen to be 2–20 times lower than that for CS-80(200)·HCl.

Pure Si(OGly)_4_ in water (pH 3.9) at room temperature gels quite slowly (point on the ordinate axis of Figure 2a). The addition of GM (pH 5.6–5.7), as observed previously [26,41,43] for PVA and CS in the salt form of acetate or ascorbate, significantly accelerates this process, and the concentration dependence of the gelation time passes through a minimum at *C*_GM_/*C*_Si_ ~0.12. It can be assumed that at low concentrations of this polysaccharide, the catalytic effect of its –OH groups predominates (as in the case of CS salts and other polysaccharides). As the concentration of GM increases, steric hindrances formed by its closely located coils for the formation of a continuous gel network (bonds ≡Si–O–Si≡) begin to increase. The two effects gradually compensate for each other, and the gelation time increases almost to the level of silicon glycerolate without GM.

The addition of CS-80·HCl to Si(OGly)_4_ + GM in a ratio of GM + CS-80·HCl = 1:1 (pH 4.4–4.5) does not lead to a significant change in the gelation time but shifts the minimum on the curve towards high concentrations of the polymer substance (Figure 2a). At a ratio of GM + CS-80·HCl = 1:2 (pH 4.2–4.4), the gelation time significantly decreased at low *C*_Polym_/*C*_Si_, and the minimum gelation time was observed at almost the same values of *C*_Polym_/*C*_Si_ ~0.10 as for GM. Probably, in the system with a higher chitosan content (with an equal mass of the polymer substance), compacted mixed associates of GM with CS-80·HCl create the most effective conditions for catalyzing the hydrolysis of the precursor (Si(OGly)_4_) and the formation of a 3D spatial network (≡Si–O–Si≡), while steric hindrances at high *C*_Polym_/*C*_Si_ are unlikely to change much.

Based on the experiments, it appears that in the presence of GM and CS-80(200)·HCl, the polycondensation mechanism of the interaction of Si(OGly)_4_ with H_2_O described in [38] and consisting of the partial hydrolysis of Si–O–C bonds with the formation of silanol groups Si–OH and their subsequent condensation into a 3D network structure of Si–O–Si bonds, does not change. However, the sol–gel reaction rate increases significantly, leading to a decrease in the gelation time of the multicomponent polysaccharide-containing system by more than 4 times compared to Si(OGly)_4_.

The addition of AmA increased the pH of the gel-forming mixture up to 5.1–5.3 and accelerated gelation even more, especially in the range of small *C*_Polym_/*C*_Si_ ratios (Figure 2a). This can be explained by the buffering effect of this amino acid and the shift of pH towards higher values. Since AmA is a weaker acid than HCl and does not interact with CS·HCl (verified by potentiometric titration), an increase in pH accelerates the silanol condensation of silicon tetraglycerolate due to a shift in the acid-base balance, redistribution of protons between the macromolecular coil of chitosan and the bulk of the system and, as a result, compaction of macrochain associates. In this regard, the gelation of the mixed composition as a whole is accelerated. A similar effect of AmA on the intensification of gelation was also observed in the mixed compositions of GM with the high-molecular-weight CS-200·HCl.

An even more pronounced effect of accelerating gelation is achieved by adding sodium tetraborate (pH 6.2–6.3). It is an effective cross-linking agent for diol fragments and is, therefore, capable of strengthening the spatial matrix of the hydrogel. Therefore, it was not surprising that the gelation time of the Si(OGly)_4_ + GM + CS-80(200)·HCl + AmA + STB systems did not exceed 20 min (a reduction by an order of magnitude or lower compared to the system without STB, Figure 2b). A gelation time of even shorter than 2 min was achieved. The high molecular weight of chitosan facilitates the process due to the greater availability of its hydroxyl groups. The slowdown in gelation with increasing *C*_Polym_/*C*_Si_ ratio (the increasing nature of the curves in Figure 2b) confirms that it is a polysiloxane gel which is formed, in whose structure GM and CS-80(200)·HCl remain in the form of macromolecular aggregates.

It should be noted that the observed gelation times (4–5 min or shorter) are achieved under mild conditions (25 °C) without additional heating of the mixed composition.

### 2.3. Effect of Medium Acidity on the Gelation Time of the System Based on Silicon Tetraglycerolate, Chitosan Hydrochloride and Glucomannan

**Figure 3:**
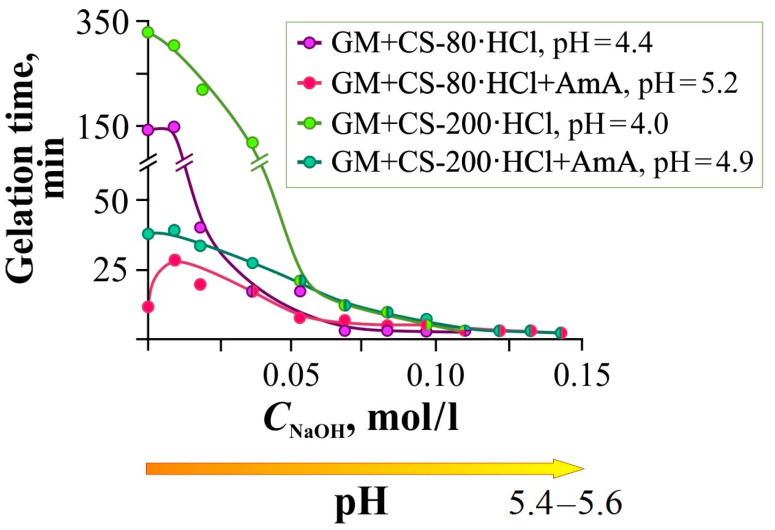
Dependence of the gelation time of the GM + CS-80(200)·HCl mixture with and without the addition of AmA on the NaOH concentration in the gel-forming system, 25 °C. The pH values in the legend correspond to the acidity index of the initial system before the introduction of NaOH. The absolute concentrations of the polymers and NaOH in the mixture are given in Table S4.

The pH shift toward higher values, achieved by adding AmA with the effect of an acid-base buffer, can be enhanced by adding alkali. While AmA causes a soft buffer shift of the acid-base equilibrium in the system without directly interacting with its components, a strong alkali with a high concentration of OH^−^ ions can cause direct deprotonation of the amino groups of chitosan, which should further accelerate gelation. Figure 3 shows the dependence of the gelation time of the Si(OGly)_4_ + GM + CS-80(200)·HCl mixtures with and without the addition of AmA on the NaOH concentration in the system.

NaOH addition to the gel-forming composition reduced the gelation time down to 2–4 min (Figure 3, pH = 5.2–5.6). This effect was observed to the greatest extent in the presence of AmA, which increases the pH of the initial mixture. According to Khonina et al. [38], in the pH range of 4–7, the gelation time of Si(OGly)_4_ is approximately the same. Therefore, the observed effect is due to the influence of alkali on chitosan hydrochloride with partial deprotonation of amino groups and strengthening of intermolecular hydrogen bonding, as well as the catalytic effect of compacted macroassociates. The curves for CS-200·HCl lie higher than for CS-80·HCl, which is possibly due to the already mentioned difference in the content of bound hydrochloric acid in these samples.

It should be noted that alkali, like STB, significantly reduces the gelation time, but, unlike STB, does not compact the glycerohydrogel structure: the introduction of STB is accompanied by additional cross-linking of the diol fragments of the polysaccharide chains, mainly GM. This expands the range of practical applications of the hybrid glycerohydrogels studied.

It can be concluded that of the two polymers present in the gel-forming system, chitosan is most susceptible to the influence of acid-base additives due to the charged nature of its macromolecules. Therefore, the next section is devoted to experiments with CS-80(200)·HCl.

### 2.4. Effect of Temperature and Functional Additives on the Gelation Time in the System Based on Silicon Glycerolate and Chitosan Hydrochloride

**Figure 4:**
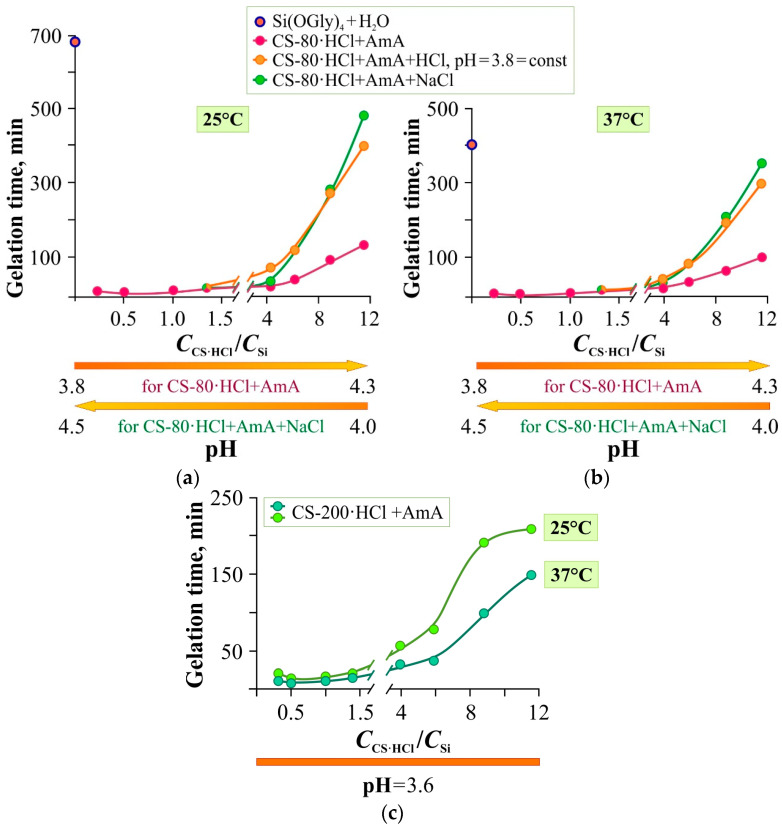
Dependence of the gelation time of the systems based on CS-80·HCl + AmA (**a**,**b**) and CS-200·HCl + AmA (**c**) without and with the addition of HCl or NaCl on the *C*_CS·HCl_/*C*_Si_ ratio, 25 °C (**a**,**c**) and 37 °C (**b**,**c**). The absolute concentrations of the polymers in the mixture are given in Table S5.

For a more detailed understanding of the effect of functional additives on the gelation time of GM + CS-80(200)·HCl mixtures, studies similar to those described above were conducted for systems with individual chitosan hydrochloride as a template, since this polymer is more responsive to changes in environmental conditions (in particular, pH). Compositions with the AmA additive were used, which showed the shortest gelation times. NaCl was additionally used as a functional additive, it accelerates gelation, according to Khonina et al. [38], leading to an increased pH, apparently due to an increased ionic strength of the environment. The pH of the system was varied by introducing HCl (the acid-solvent for both chitosan samples). The temperature range of the sol–gel synthesis was expanded (25 and 37 °C, physiological temperature). Figure 4 illustrates the effect of temperature, as well as the *C*_Polym_/*C*_Si_ ratio in the system, the average molecular weight of chitosan hydrochloride and low-molecular-weight additives on the gelation time.

As noted in Section 2.2, pure silicon glycerolate gelates very slowly (~700 min at 25 °C and ~400 min at 37 °C, pH 3.9). When adding GM or a GM + CS·HCl mixture, which first acts as a catalyst for the hydrolysis of the precursor and polycondensation, and then as a template, the gelation time dropped sharply, passed through a minimum, and then increased (Figure 2a). Obviously, before the minimum, the main influence is exerted by the increase in the concentration of –OH groups exhibiting a catalytic effect. After the minimum, steric difficulties created by template coils on the path of the forming network of polyorganosiloxane gel increase.

This pattern of the influence of polymer concentration on the gelation kinetics was also observed for the compositions based on individual chitosan hydrochloride: both low-molecular-weight CS-80·HCl (Figure 4a,b) and high-molecular-weight CS-200·HCl (Figure 4c). However, in the latter case, the gelation times were somewhat higher. This is explained by the fact that larger coils (see Figure 1c, Table 1) create more steric hindrance for the growing network of silanol gel.

At low *C*_CS·HCl_/*C*_Si_ ratios, hydrochloric acid, as well as the neutral salt sodium chloride, have virtually no effect on the rate of gelation (Figure 4a,b). In this region, as already mentioned, the catalytic effect of –OH groups of the aminopolysaccharide on the hydrolysis of the precursor and polycondensation prevails. It would seem that the introduction of these additives should lead to multidirectional effects, since HCl causes additional protonation of amino groups, whilst NaCl, on the contrary, neutralizes macromolecular charges. It should be noted that, according to Czechowska-Biskup et al. [52], hydrochloric acid introduced into a CS solution in excess of its stoichiometric amount could play the role of a neutral electrolyte (like NaCl), enhancing the screening of charged amino groups and weakening the polyelectrolyte effect. This explains the virtually identical effect of HCl and NaCl on gelation kinetics. It should be assumed that the amino groups of chitosan do not have the same catalytic effect as hydroxyl groups, and the conformational changes caused by the compression of macroions both upon the addition of HCl and with the increase in ionic strength upon the addition of NaCl have virtually no effect on the availability of hydroxyl groups for foreign particles.

At the same time, at high *C*_CS·HCl_/*C*_Si_ ratios, both HCl and NaCl significantly slow down gelation (Figure 4a,b). This is already the region of prevalence of steric hindrance. Since in this region of the compositions of the gelling mixture *C*_CS·HCl_ is significantly higher than *C*_HCl_, when adding hydrochloric acid. The observed effect can be explained by additional protonation of the amino groups of chitosan with an increase in the polyelectrolyte effect and swelling of the macrocoils of the polyelectrolyte template. Consequently, the conformational changes caused by protonation (stretching of macroions) create more obstacles to the formation of a continuous network of silanol bonds. The slowdown in gelation upon NaCl addition could also be caused by a shift in the acid-base equilibrium since a decrease in pH from 4.5 to 4.0 was noted. However, this is somewhat unexpected, since the electrolyte salts of the lyotropic Hofmeister series accelerate the gelation of Si(OGly)_4_ [38]. The mechanism of increasing acidity in such a system is still unclear and is currently being studied, but the effect of increased acidity is indicated above.

Note also that with increasing temperature (25 °C → 37 °C, Figure 4), gelation accelerates for both CS·HCl samples and all functional additives, since the rate of precursor hydrolysis and polycondensation reactions increases. Acceleration of gelation at physiological temperature is very promising in practical terms, for example, for the design of liquid patches, one of the main requirements for which is rapid gelation on the dermal surface of a living organism (the dermal temperature varies from 33.5 °C to 36.9 °C).

Thus, the additional low-molecular-weight additives we tested either do not affect or slow down gelation. In general, the best additives that accelerate gelation of the Si(OGly)_4_ + GM + CS-80(200)·HCl system and structure the spatial network of glycerohydrogel are AmA and STB.

### 2.5. Mucoadhesive and Dermoadhesive Properties of the Glycerohydrogels Based on Silicon Tetraglycerolate, Chitosan and Glucomannan

**Figure 5:**
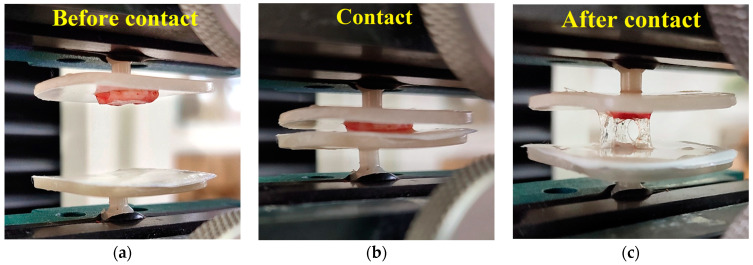
Photos of our biomedical experiments on a tensile testing machine with the obtained glycerohydrogel based on CS-80·HCl. (**a**) Fixed flap of rat skin (upper platform) and applied glycerohydrogel (lower platform). (**b**) Bringing the glycerohydrogel into contact with the dermal surface of rat skin with a force of 1.3 N. (**c**) The state of the adhesive contact at the end of the experiment to overcome adhesive forces.

Two compositions of the studied glycerohydrogels based on CS-80·HCl and CS-200·HCl with the shortest gelation times were selected for biological testing (Figure 2b; Table S3-2). Their mucoadhesive and dermoadhesive properties were examined by the peel-off method under conditions of overcoming the adhesion forces of the hydrogel preparation from the model mucin-containing substrate (in vitro) or the dermal surface of rat skin (ex vivo) (Figure 5).

The obtained values of the maximum detachment force of mucoadhesion (*W*_M_) and the maximum detachment force of dermoadhesion (*W*_D_) were compared with those for the pharmaceutical hydrogel preparation Metrogyl Denta^®^ based on Carbomer-980, a copolymer of acrylic acid (Table 2).

As can be seen from the table, the composition based on CS-80·HCl surpasses the control based on a synthetic biologically inactive polymer in mucoadhesion and is only slightly inferior to it in dermoadhesion. The composition based on CS-200·HCl surpasses the control in dermoadhesion, while its mucoadhesive properties, although lower than the control, are sufficient for short-term retention of the hydrogel on the mucous tissue (i.e., remain within the permissible limits). We can conclude that our glycerohydrogels have good mucoadhesive and dermoadhesive properties, ensuring their close contact with the wound surface, prolonging the time the glycerohydrogel remains on the wound, and also performing barrier and moisturizing functions. These factors play a significant role in the design of wound healing coatings.

It should also be noted that in vivo testing of the biological activity of our glycerohydrogels Si(OGly)_4_ + GM + CS-80(200)·HCl + AmA + STB, characterized by an ultra-short gelation time, revealed a hemostatic effect and demonstrated high wound healing capacity, which is the subject of further reports.

## 3. Conclusions

Mixtures of various compositions based on silicon teraglycerolate, glucomannan and chitosan hydrochloride (with molecular weights of 80 and 200 kDa) with a number of functional additives (aminocaproic and hydrochloric acids, sodium chloride, hydroxide and tetraborate) were prepared and tested to obtain Si–polysaccharide-containing glycerohydrogels. The best positive effect was shown by AmA, STB and NaOH, shifting the pH to neutrality and accelerating the hydrolysis and polycondensation of the sol–gel precursor. The introduction of STB also led to additional structuring of the glycerohydrogel matrix due to the cross-linking of sterically accessible diol fragments of macrochains. The accelerating effect of the additives on the formation of a 3D spatial network of ≡Si–O–Si≡ bonds is largely determined by the template-to-precursor mass ratio, the concentration of GM and CS-80(200)·HCl, the degree of compaction of their mixed macromolecular associates, the molecular weight of the aminopolysaccharide, and the temperature conditions of the sol–gel process. By varying the composition of the gelling system and introducing additives, we were able to achieve a short gelation time (5 min or shorter), which is promising in practical terms. For example, the gelation time of the composition (wt.%) 0.25 GM + 2.0 CS-80(200)·HCl + 2.0 AmA + 0.001 STB with a polymer/precursor ratio of 1.09 is only 1.5 min. Some glycerohydrogel preparations are not inferior to the commercial one Metrogyl Denta^®^ in terms of ultimate mucoadhesion (26 kPa) and dermoadhesion (16 kPa). Thus, the glycerohydrogels synthesized by us could find application in the design of wound dressings, liquid plasters, and other medical applications.

Thus, the glycerohydrogels synthesized by us show promising potential for creating wound dressings for various functional purposes and could find use in such medical applications as traumatology, surgery, regenerative medicine, dermatology, cosmetology, and dentistry. In particular, the synergistic effects of the interacting components, leading to ultra-fast gelation, are very promising for the development of new-generation liquid plasters, whose gelation should occur directly on the wound surface. The fundamental difference between such liquid plasters and commercial medical adhesives and liquid bandages based on synthetic polymers, which form a protective bioinert film coating only, is biocompatibility and a wide range of biological activities of the ingredients included in the polysaccharide-containing glycerohydrogel material. It seems that the biologically active 3D polymeric matrix filled with water and glycerin will not only protect the wound from infection but also have a multifunctional therapeutic effect. If necessary, water- or glycerol-soluble medicinal substances could be introduced into the gel-forming composition.

## 4. Materials and Methods

### 4.1. Materials

We used two powdered samples of CS-80·HCl (in the form of hydrochloride with 9.7 wt.% Cl) and CS-200 with an average viscosity molecular weight of 80 and 200 kDa, respectively, and a close degree of deacetylation of 80 ± 2 mol.% (ZAO Bioprogress, Shchyolkovo, Russia); GM with an average viscosity molecular weight of 1100 kDa (Uspekh Ltd., Saint-Petersburg, Russia) (See Figure S1 for the structural formulae); ε-AmA (Vekton Ltd., Saint-Petersburg, Russia); 0.1 N HCl (ZAO UralKhimInvest, Ufa, Russia); NaOH fixanal and NaCl of chemically pure grade (Base of Chemical Reactants No. 1 Ltd., Moscow, Russia); 58.7% glycerol solution of Si(OGly)_4_ (IOS UB RAS, Yekaterinburg, Russia); 20% glycerol solution of Na_2_B_4_O_7_ (OJSC Ivanovo Pharmaceutical Factory, Ivanovo, Russia); 96% C_2_H_5_OH (ZAO RFK, Moscow, Russia); glycerol (Chemical Reactant Base No. 1 Ltd., Vyazma, Russia) and double distilled water (ddw).

#### 4.1.1. Preparation of Solutions

The methods for preparing solutions of low- and high-molecular-weight chitosan hydrochlorides differed. Solutions of low-molecular-weight CS-80·HCl were prepared by dissolving a weighed portion of the polymer in a calculated amount of ddw (solvent) under stirring. Solutions of high-molecular-weight CS-200·HCl were prepared by dispersing a weighed portion of CS-200 in a calculated amount of ddw (non-solvent) for 30 min, followed by the addition of 0.1 N hydrochloric acid (in an equimolar ratio to the amino groups of CS-200) under continuous stirring until complete dissolution. Taking into account the protonation degree of amino groups, which is 0.9 under such conditions, the Cl content in the CS-200·HCl sample was 7.6 wt.%. The obtained CS-80(200)·HCl solutions were kept at 20 ± 2 °C for 1 day. Additives of aqueous solutions of AmA (*C*_AmA_ = 1.0–4.0 g·dL^−1^) and NaCl (*C*_NaCl_ = 1.0–4.0 g·dL^−1^), glycerol solution of STB (*C*_STB_ = 10 g·dL^−1^), 0.1 N NaOH and 0.1 N HCl were introduced into the finished CS-80(200)·HCl solution. The lower concentration value of the additives introduced was due to the absence of any significant modifying effect, the higher one being due to the precipitation of the polymer before the onset of gelation. The initial concentration of the CS-80(200)·HCl solution for viscometric studies and for studying the gelation properties was *C*_CS·HCl_ = 0.06–0.1 g·dL^−1^ and 1.0–4.0 g·dL^−1^, respectively.

To obtain GM solutions, the calculated amount of H_2_O was added to the alcohol suspension of the polymer (volume ratio GM:C_2_H_5_OH = 1.0:0.5) and stirred on a magnetic stirrer at a rotation speed of 500–600 rpm at 25 °C for 10 min, followed by heating up to ~80 °C using microwave radiation with a power of 800 W (4 times for 30 s) to remove ethyl alcohol. The initial concentration of the GM solution for viscometric studies and for studying the gelling properties was *C*_GM_ = 0.1 g·dL^−1^ and 0.2 or 0.5 g·dL^−1^, respectively.

#### 4.1.2. Preparation of Glycerohydrogels

Glycerohydrogels were prepared by mixing the initial aqueous solutions of GM, CS-80(200)·HCl or their mixtures with a glycerol solution of Si(OGly)_4_ with and without the addition of AmA, STB, NaOH, HCl and NaCl in various weight ratios. The absolute concentrations of the polymers in the gel-forming composition and their values in terms of the silicon concentration in the sol–gel precursor are given in Tables S3–S5. The resulting systems were homogenized by stirring with a glass rod for 1 min and left under static conditions at 24 ± 2 or 37 ± 0.2 °C for the gelation process to proceed. The sol–gel transition time was recorded based on the time of loss of system fluidity from the moment of mixing the components using the “flask inversion” method with an accuracy of 3–5 s in the case of rapid sol–gel synthesis and 3–5 min for long gelation times.

### 4.2. Methods

#### 4.2.1. Gravimetric Method

The sample weight was controlled gravimetrically on an Ohaus Adventurer AR 1530 analytical balance, weighing accuracy ±0.002 g. To express the component composition of the mixture, the template/precursor mass ratio (*C*_Polym_/*C*_Si_) was used, where “Polym” corresponds to the total mass concentration of GM and CS·HCl in the polymer mixture.

#### 4.2.2. Viscometric Method

Viscometric studies were carried out in an Ubbelohde viscometer (RF) with a capillary diameter of 0.56 mm at 25 ± 0.1 °C using the generally accepted method. Immediately before the measurements, the solutions were filtered through a No. 160 Schott glass filter (Russian Chemist Ltd., Moscow, Russia). In the case of a linear concentration dependence of the viscosity number (η_sp_/*C*, dL/g; η_sp_ is specific viscosity), the intrinsic viscosity ([η], dL/g) was determined using the Huggins equation η_sp_/*C* = [η] + *K*′[η]^2^*C* (*C* is concentration, g/dL), and in the case of a curvilinear one we used the equation [η] = (∂ln η_red_/∂*C*)_C→0_ (η_rel_ = η/η_0_ is relative viscosity, where η and η_0_ is the viscosity of the solution and solvent, respectively; η_sp_ = η_rel_ − 1) [51].

The additive intrinsic viscosities [η]_add_ were calculated by the formula:

$${\left[\eta \right]}_{add}=\left(1-\frac{Ratio}{100}\right){\left[\eta \right]}_{GM}+\frac{Ratio}{100}\;{\left[\eta \right]}_{CS},$$

where [η]_GM_ and [η]_CS_ are the intrinsic viscosities of GM and CS, respectively, Ratio is the CS to GM + CS weight ratio at any dilution used.

In the case of chitosan samples, the molecular weight value provided by the manufacturer was used. In the case of GM, the average molecular weight was estimated viscosimetrically in separate experiments and calculated using the Kuhn–Mark–Houwink formula with the parameters by Tatirat et al. [53]:

$${\bar{M}}_{\eta }={\left(\frac{\left[\eta \right]}{6.37\times 10^{-4}}\right)}^{1/0.74}.$$

The effective radius $R_{ef}$ of the macromolecular coil at infinite dilution was calculated using the Flory–Fox formula:

(1) $$R_{ef}=\sqrt[{3}]{10\pi \frac{\left[\eta \right]{\bar{M}}_{\eta }}{N_{A}}},$$

where ${\bar{M}}_{\eta }$ is the molecular weight, kDa; $N_{A}$ Avogadro’s number, mol^−1^.

#### 4.2.3. Physicomechanical Tests

The mucoadhesive and dermoadhesive properties of our glycerohydrogels were assessed using a Tinius Olsen H1KS tensile testing machine (England) using the tear-off method by measuring the maximum force to be applied to overcome the adhesive force in the contact zone of the glycerohydrogel preparation with a model substrate simulating in vitro mucous or ex vivo dermal tissue. To form an adhesive contact along the glycerohydrogel–substrate interface, the glycerohydrogel surface was brought into contact with the substrate with a force of 1.3 N for 2 min. Then, a tearing force was applied at a constant speed of 0.1 mm/s until the glycerohydrogel was completely separated from the substrate. The maximum detachment forces of mucoadhesion (*W*_M_, kN/m^2^) and dermoadhesion (*W*_D_, kN/m^2^) were determined as the force required to destroy the adhesive bond, divided by the area of the interphase boundary. At least 10 replicate experiments were conducted.

Mucin with 99% basic substance—mucous secretion filtrate, Helix Aspersa Snail (La Coruña, Spain)—was used as a mucoadhesive material. A rat skin flap was used as a dermoadhesive substrate. Dermis samples were obtained by excising the skin of the withers of rats with longitudinal incisions and cutting out a 1 × 1 cm flap; the skin was removed down to the fascia. The rats were provided by the vivarium of the Saratov State Medical University named after V.I. Razumovsky.

## Figures and Tables

**Table 1:** Intrinsic viscosity [η] and effective hydrodynamic radius *R*_ef_ of the polymers (GM, CS-80·HCl, and CS·200·HCl).

Polymer	[η], dL/g	*R*_ef_, nm
GM	20.0	70 ± 2
CS-80 HCl	21.7	30 ± 1
CS-200 HCl	23.7	40 ± 1

**Table 2:** Adhesive properties of our glycerohydrogels Si(OGly)_4_ + GM + CS-80(200)·HCl + AmA + STB with the shortest gelation times (Figure 2b, marked with dotted lines; Table S3-2).

Commercial Hydrogel Preparation/Main Component of the Glycerohydrogel		Gelation Time, min	Maximum Detachment Force, kN/m^2^	
			of Mucoadhesion (*W*_M_)	of Dermoadhesion (*W*_D_)
«Metrogyl Denta»™	Control	–	21.9 ± 1.3	13.2 ± 0.7
CS-80·HCl	See Table S3-2 for composition	4.5	25.0 ± 1.5	11.7 ± 0.6
CS-200·HCl		1.5	12.4 ± 0.7	16.2 ± 0.8

## Data Availability

Data are contained within the article and Supplementary Material.

## Supplementary Materials

The following supporting information can be downloaded at: https://www.mdpi.com/article/10.3390/gels11020103/s1, Figure S1: Structural formulae of chitosan hydrochloride (**a**) and glucomannan (**b**) in powder form; Figure S2: Concentration dependences of the viscosity of aqueous solutions: (**a**) CS-80·HCl: GM in the ratio of components 1:9 wt.% in the Huggins coordinates η_sp_/*C* = [η] + *K*′[η]^2^*C*; (**b**,**c**) CS-80(200)·HCl and CS-80(200)·HCl: GM = 1:1 – 9:1 wt.% in the ln η_rel_ = *f*(*C*) coordinates, 25 °C; Table S3-1: Composition and characteristics of the gelling mixtures GM + Si(OGly)_4_ and GM + CS-80·HCl + Si(OGly)_4_ with and without AmA; Table S3-2. Composition and characteristics of the gelling mixtures GM + CS-80·HCl + Si(OGly)_4_ with the addition of AmA and STB; Table S4. Composition and characteristics of the gelling mixtures GM + CS-80·HCl + Si(OGly)_4_ and GM + CS-80·HCl + AmA + Si(OGly)_4_ with the addition of NaOH; Table S5. Composition and characteristics of the gelling mixtures CS-80·HCl + AmA + Si(OGly)_4_ with the addition of HCl or NaCl.
