# Supplementary material for: Preparation and Properties of Glycerohydrogels Based on Silicon Tetraglycerolate, Chitosan Hydrochloride and Glucomannan
Source: Gels. 2025 Feb 2;11(2):103. doi: 10.3390/gels11020103 (PMC11854711; doi:10.3390/gels11020103)
Supplement: Supplementary file 1 [file gels-11-00103-s001.zip › gels-3405877-supplementary.pdf]

Supporting Information

Figure S1. Structural formulae of chitosan hydrochloride (a) and glucomannan (b) in powder form

(a) (b)

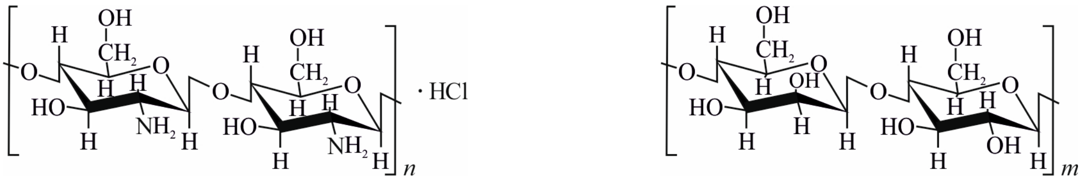

Figure S2. Viscometric properties of solutions of glucomannan, chitosan hydrochloride and their mixtures

(a) (b) (c)

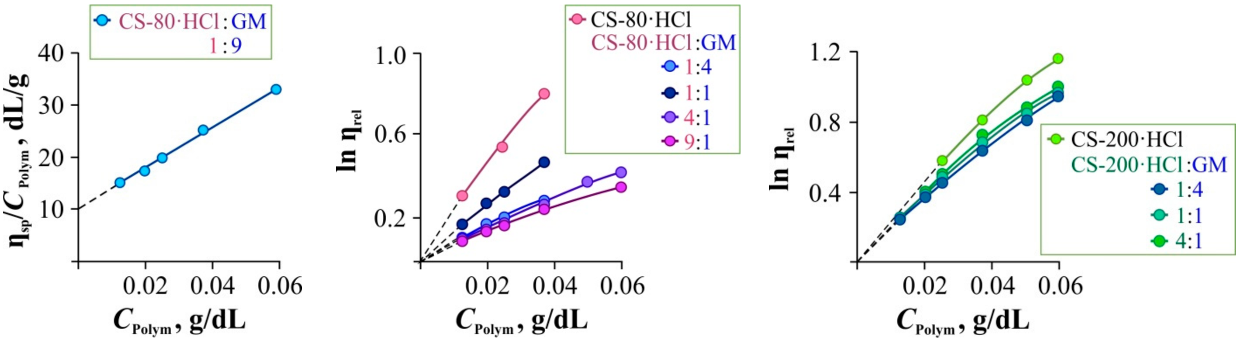

Concentration dependences of the viscosity of aqueous solutions: (a) – CS-80·HCl : GM in the ratio of components 1:9 wt.% in the Huggins coordinates $\eta_{sp}/C = [\eta] + K'[\eta]^2C$; (b) and (c) – CS-80(200)·HCl and CS-80(200)·HCl : GM = 1:1 – 9:1 wt.% in the ln $\eta_{rel} = f(C)$ coordinates, 25°C.

S3. Influence of functional additives on the gelation time of the system based on silicon tetraglycerolate, chitosan hydrochloride and glucomannan

Table S3-1. Composition and characteristics of the gelling mixtures GM + $Si(OGly)_4$ and GM + CS-80·HCl + $Si(OGly)_4$ with and without AmA

Composition	Ratio Polym/$Si(OGly)_4$	Ratio GM : CS-80·HCl	Concentration of the initial solutions, wt.%				Mass content of the components, wt.%					pH	Gelation time, min
			$C_{CS·HCl}$	C_{GM}	C_{AmA}	C_{Si}	$C_{CS·HCl}$	C_{GM}	C_{AmA}	C_{Si}	C_{Polym}/C_{Si}		
GM + $Si(OGly)_4$	1/4	–	–	0.25	–	4.12	–	0.05	–	3.30	0.02	5.6–5.7	365
	1/2							0.08		2.75	0.03		271
	1/1							0.13		2.06	0.06		179
	2/1							0.17		1.37	0.12		141
	4/1							0.20		0.82	0.24		268
	6/1							0.21		0.59	0.36		598
GM + CS-80·HCl + $Si(OGly)_4$	1/4	1:1	1.0				0.20	0.05		3.30	0.08	4.4–4.5	320
	1/2						0.33	0.08		2.75	0.15		240
	1/1						0.50	0.13		2.06	0.30		200
	2/1						0.67	0.17		1.37	0.61		180
	4/1						0.80	0.20		0.82	1.21		306
	6/1						0.86	0.21		0.59	1.82		450
GM + CS-80·HCl + $Si(OGly)_4$	1/4	1:2					0.13	0.03		3.30	0.05	4.2–4.4	265
	1/2						0.22	0.05		2.75	0.10		148
	1/1						0.33	0.08		2.06	0.20		150
	2/1						0.45	0.11		1.37	0.40		165
	4/1						0.54	0.13		0.82	0.81		300
	6/1						0.57	0.14		0.59	1.21		490
GM + CS-80·HCl + AmA + $Si(OGly)_4$	1/4	1:1			1.0		0.20	0.05	0.20	3.30	0.08	5.30	32
	1/2						0.33	0.08	0.33	2.75	0.15	5.35	15
	1/1						0.50	0.13	0.50	2.06	0.30	5.28	25
	2/1						0.67	0.17	0.67	1.37	0.61	5.20	40
	4/1						0.80	0.20	0.80	0.82	1.21	5.18	180
	6/1						0.86	0.21	0.86	0.59	1.82	5.15	300
GM + CS-80·HCl + AmA + $Si(OGly)_4$	1/4	1:2					0.13	0.03	0.13	3.30	0.05	5.25	30
	1/2						0.22	0.05	0.22	2.75	0.10	5.20	14
	1/1						0.33	0.08	0.34	2.06	0.20	5.17	18
	2/1						0.45	0.11	0.45	1.37	0.40	5.15	34
	4/1						0.54	0.13	0.54	0.82	0.81	5.13	145
	6/1						0.57	0.14	0.57	0.59	1.21	5.11	205

Table S3-2. Composition and characteristics of the gelling mixtures GM + CS-80·HCl + $Si(OGly)_4$ with the addition of AmA and STB

<table>
<tr><th rowspan="2">Composition</th><th rowspan="2">Ratio Polym/$Si(OGly)_4$</th><th colspan="5">Concentration of the initial solutions, wt.%</th><th colspan="6">Mass content of the components, wt.%</th><th rowspan="2">pH</th><th rowspan="2">Gelation time, min</th></tr>
<tr><th>$C_{CS·HCl}$</th><th>C_{GM}</th><th>C_{AmA}</th><th>C_{STB}</th><th>C_{Si}</th><th>$C_{CS·HCl}$</th><th>C_{GM}</th><th>C_{AmA}</th><th>C_{STB}</th><th>C_{Si}</th><th>C_{Polym}/C_{Si}</th></tr>
<tr><td rowspan="3">GM + CS-80·HCl + AmA + $Si(OGly)_4$</td><td>1/1</td><td rowspan="6">4.0</td><td rowspan="6">0.5</td><td rowspan="6">4.0</td><td rowspan="6">10</td><td rowspan="6">4.12</td><td>**2.0**</td><td>**0.25**</td><td>**2.0**</td><td rowspan="3">**0.001**</td><td>**2.06**</td><td>**1.09**</td><td rowspan="3">6.3–6.4</td><td>**4.5***</td></tr>
<tr><td>1.5/1</td><td>2.4</td><td>0.30</td><td>2.4</td><td>1.65</td><td>1.64</td><td>10.5</td></tr>
<tr><td>2/1</td><td>2.7</td><td>0.33</td><td>2.7</td><td>1.37</td><td>2.21</td><td>20.0</td></tr>
<tr><td rowspan="3">GM + CS-200·HCl + AmA + $Si(OGly)_4$</td><td>1/1</td><td>**2.0**</td><td>**0.10**</td><td>**2.0**</td><td rowspan="3">**0.001**</td><td>**2.06**</td><td>**1.02**</td><td rowspan="3">6.2–6.3</td><td>**1.5***</td></tr>
<tr><td>1.5/1</td><td>2.4</td><td>0.12</td><td>2.4</td><td>1.65</td><td>1.53</td><td>6.0</td></tr>
<tr><td>2/1</td><td>2.7</td><td>0.13</td><td>2.7</td><td>1.37</td><td>2.07</td><td>15.0</td></tr>
</table>

*Super-fast gelation systems used to evaluate their mucoadhesive and dermoadhesive properties

S4. Influence of medium acidity on the gelation time of the system based on silicon tetraglycerolate, chitosan hydrochloride and glucomannan

Table S4. Composition and characteristics of the gelling mixtures GM + CS-80·HCl + $Si(OGly)_4$ and GM + CS-80·HCl + AmA + $Si(OGly)_4$ with the addition of NaOH

Composition	Ratio Polym/$Si(OGly)_4$	Ratio GM : CS-80·HCl	Concentration of the initial solutions, wt.%					Mass content of the components, wt.%						pH	Gelation time, min
			$C_{CS·HCl}$	C_{GM}	C_{NaOH}	C_{AmA}	C_{Si}	$C_{CS·HCl}$	C_{GM}	C_{NaOH}	C_{AmA}	C_{Si}	C_{Polym}/C_{Si}		
GM + CS-80·HCl + $Si(OGly)_4$	1/1	1:2	1.0	0.5	–	–	4.12	0.33	0.08	–	–	2.06	0.20	4.37	140
					0.032					0.001				4.24	150
					0.093					0.003				4.34	40
					0.153					0.005				5.41	17
					0.211					0.007				4.60	7
					0.267					0.009				4.79	3
					0.322					0.010				4.94	3
					0.374					0.012				5.24	2.5
					0.426					0.014				5.44	2.2
GM + CS-80·HCl + AmA + $Si(OGly)_4$	1/1				–	1.0				–	0.33			5.20	12
					0.032					0.001				4.80	28
					0.093					0.003				4.99	20
					0.153					0.005				5.12	17
					0.211					0.007				5.14	8
					0.267					0.009				5.23	7
					0.322					0.010				5.34	5
					0.374					0.012				5.36	4
					0.426					0.014				5.41	3
					0.476					0.016				5.47	3
					0.524					0.018				5.51	3
					0.572					0.020				5.63	3
GM + CS-200·HCl + $Si(OGly)_4$	1/1				–	–				–	–			4.02	330
					0.032					0.001				4.11	300
					0.093					0.003				4.28	200
					0.153					0.005				4.55	120
					0.211					0.007				4.81	21
					0.267					0.009				5.01	12
					0.322					0.010				5.20	10
					0.374					0.012				5.31	5
					0.426					0.014				5.43	3
GM + CS-200·HCl + AmA + $Si(OGly)_4$	1/1				–	1.0				–	0.33			4.88	37
					0.032					0.001				4.80	39
					0.093					0.003				4.85	34
					0.153					0.005				4.92	27
					0.211					0.007				4.93	21
					0.267					0.009				5.01	12
					0.322					0.010				5.10	10
					0.374					0.012				5.13	7
					0.426					0.014				5.17	3
					0.476					0.016				5.20	3
					0.524					0.018				5.42	3
					0.572					0.020				5.47	1.8

S5. Influence of temperature and functional additives on the gelation time in the system based on silicon tetraglycerolate and individual chitosan hydrochloride

Table S5. Composition and characteristics of the gelling mixtures CS-80(200)·HCl + AmA + $Si(OGly)_4$ with the addition of HCl or NaCl

Composition	Ratio Polym/$Si(OGly)_4$	Concentration of the initial solutions, wt.%					Mass content of the components, wt.%				pH	Gelation time, min, Temperature, °C	
		$C_{CS·HCl}$	C_{AmA}	C_{HCl}	C_{NaCl}	C_{Si}	$C_{CS·HCl}$	C_{AmA}	C_{Si}	C_{Polym}/C_{Si}		25	37
CS-80·HCl + AmA + $Si(OGly)_4$	1/4	4.0	4.0	–	–	4.12	0.80	0.80	3.30	0.24	4.13	–	–
	1/2						1.33	1.33	2.75	0.48	3.76	11	7
	1/1						2.00	2.00	2.06	0.98	3.95	8	5
	2/1						2.67	2.67	1.37	1.37	3.87	13	7
	4/1						3.20	3.20	0.82	3.90	4.03	17	14
	6/1						3.45	3.45	0.59	5.89	4.13	22	19
	9/1						3.60	3.60	0.41	8.78	4.21	38	28
	12/1						3.69	3.69	0.32	11.53	4.27	94	65
CS-80·HCl + AmA + HCl + $Si(OGly)_4$	2/1			3.7			2.67	2.67	1.37	1.37	3.80	25	17
	4/1						3.20	3.20	0.82	3.90		70	45
	6/1						3.45	3.45	0.59	5.89		120	80
	9/1						3.60	3.60	0.41	8.78		260	190
	12/1						3.69	3.69	0.32	11.53		400	300
CS-80·HCl + AmA + NaCl + $Si(OGly)_4$	2/1			–	0.17		2.67	2.67	1.37	1.37	4.33	20	16
	4/1						3.20	3.20	0.82	3.90	4.30	40	30
	9/1						3.60	3.60	0.41	8.78	4.18	280	210
	12/1						3.69	3.69	0.32	11.53	4.08	480	360
CS-200·HCl + AmA + $Si(OGly)_4$	1/4			–	–		0.80	0.80	3.30	0.24	3.62	20	11
	1/2						1.33	1.33	2.75	0.48	3.64	14	6
	1/1						2.00	2.00	2.06	0.98	3.62	15	8
	2/1						2.67	2.67	1.37	1.37	3.60	19	13
	4/1						3.20	3.20	0.82	3.90	3.60	60	30
	6/1						3.45	3.45	0.59	5.89	3.60	76	35
	9/1						3.60	3.60	0.41	8.78	3.65	187	100
	12/1						3.69	3.69	0.32	11.53	3.63	210	150
